# The Impact of Spatial Normalization Strategies on the Temporal Features of the Resting-State Functional MRI: Spatial Normalization Before rs-fMRI Features Calculation May Reduce the Reliability

**DOI:** 10.3389/fnins.2019.01249

**Published:** 2019-11-26

**Authors:** Zhao Qing, Xin Zhang, Meiping Ye, Sichu Wu, Xin Wang, Zuzana Nedelska, Jakub Hort, Bin Zhu, Bing Zhang

**Affiliations:** ^1^Department of Radiology, The Affiliated Drum Tower Hospital of Nanjing University Medical School, Nanjing, China; ^2^Institute for Brain Sciences, Nanjing University, Nanjing, China; ^3^International Clinical Research Center, St. Anne’s University Hospital Brno, Brno, Czechia; ^4^Memory Clinic, Department of Neurology, Second Faculty of Medicine Charles University and Motol University Hospital, Prague, Czechia

**Keywords:** spatial normalization, resting-state fMRI, fMRI methods, reliability, fMRI preprocessing

## Abstract

Previous resting-state functional magnetic resonance imaging (rs-fMRI) studies frequently applied the spatial normalization on fMRI time series before the calculation of temporal features (here referred to as “Prenorm”). We hypothesized that calculating the rs-fMRI features, for example, functional connectivity (FC), regional homogeneity (ReHo), or amplitude of low-frequency fluctuation (ALFF) in individual space, before the spatial normalization (referred to as “Postnorm”) can be an improvement to avoid artifacts and increase the results’ reliability. We utilized two datasets: (1) simulated images where temporal signal-to-noise ratio (tSNR) is kept a constant and (2) an empirical fMRI dataset with 50 healthy young subjects. For simulated images, the tSNR is constant as generated in individual space but increased after Prenorm and intersubject variability of tSNR was induced. In contrast, tSNR was kept constant after Postnorm. Consistently, for empirical images, higher tSNR, ReHo, and FC (default mode network, seed in precuneus) and lower ALFF were found after Prenorm compared to those of Postnorm. Coefficient of variability of tSNR and ALFF was higher after Prenorm compared to those of Postnorm. Moreover, the significant correlation was found between simulated tSNR after Prenorm and empirical tSNR, ALFF, and ReHo after Prenorm, indicating algorithmic variation in empirical rs-fMRI features. Furthermore, comparing to Prenorm, ALFF and ReHo showed higher intraclass correlation coefficients between two serial scans after Postnorm. Our results indicated that Prenorm may induce algorithmic intersubject variability on tSNR and reduce its reliability, which also significantly affected ALFF and ReHo. We suggest using Postnorm instead of Prenorm for future rs-fMRI studies using ALFF/ReHo.

## Introduction

Resting-state functional magnetic resonance imaging (rs-fMRI), which uses blood oxygenation level-dependent (BOLD) signal to measure spontaneous brain activity, has been developed and widely applied in neuroscience and clinical research studies (Raichle, 2010; Zhang and Raichle, 2010; Haak et al., 2018). However, the reliability of rs-fMRI indices was recently concerned and challenged (Xing, 2018; Zuo et al., 2019a, b). Low reliability led to the demands for huge sample size or large effect size to achieve enough statistical power, which were both difficult for rs-fMRI studies. Therefore, it is important to find an optimized data processing strategy that can give out higher reliability of rs-fMRI features (Zuo et al., 2019b).

A typical data processing step in rs-fMRI is spatial normalization, which achieves between-subject comparison in voxel level but may also induce spatial–temporal correlation and resampling errors (Worsley, 2005; Wu et al., 2011). To date, how to estimate precise spatial transformation from an individual space into standard space has been widely discussed (Collins et al., 1994; Mazziotta et al., 2001; Tzourio-Mazoyer et al., 2002; Calhoun et al., 2017). Besides, Wu et al. (2011) compared two normalization strategies about its order in preprocessing. One is “Prenorm,” which means performing the spatial normalization on each fMRI image of each time point into standard space. Then calculate the certain temporal feature of the time series, for example, functional connectivity (FC) (Biswal et al., 1995; Fox et al., 2005; Raichle, 2015), regional homogeneity (ReHo) (Zang et al., 2004; Jiang and Zuo, 2016), or amplitude of low-frequency fluctuation (ALFF) (Zang et al., 2007; Zuo et al., 2013). This Prenorm strategy has been widely used in large portion of fMRI studies from a decade ago until recently (Fox et al., 2005; Cooper et al., 2017; Archer et al., 2018) and was the default option in several popular pipeline tools like DPABI and GRENTA (Yan and Zang, 2010; Wang et al., 2015; Yan et al., 2016). The other is “Postnorm,” which is to first calculate these feature maps in individual space, and then perform the normalization. Wu et al. (2011) reported a slight difference of the value of FC and ALFF between such two strategies, and notably, they also found that Prenorm strategy may increase intersubject variability of ALFF in individual space, but Postnorm did not. Putatively, larger variability may lead to lower reliability and indicated that widely used Prenorm may be a worse choice than Postnorm. However, it is still unclear if this effect is an algorithmic bias and if and how it may affect the reliability of rs-fMRI features.

In the current study, we aim to systemically investigate and compare the Prenorm and Postnorm strategies: (1) To verify if it is a pure algorithmic effect, we investigate if and how normalization can induce across-subject variation on a simulated dataset, which has a constant temporal signal-to-noise ratio (tSNR) in individual space for all subjects and all voxels. (2) To investigate if and how the value and intersubject variability of tSNR as well as commonly used rs-fMIR indices including ALFF, FC, and ReHo were different under different normalizations. Additionally, if the intersubject variability in empirical images affected algorithmic effect. (3) To investigate the test–retest reliability of ALFF, ReHo, and FC in an empirical fMRI dataset.

## Materials and Methods

### Data Acquisition

#### Empirical Images

We downloaded one subset of the public database “Consortium for Reliability and Reproducibility, CORR,” which is named “BNU1” (Zuo et al., 2014). The downloading Web address can be found at http://fcon_1000.projects.nitrc.org/indi/CoRR/html/_static/downloads.html. There were 57 healthy young subjects (male/female: 30/27; age: 19–30 years) in this data cohort. For each subject, there were two serial scans, which were acquired within the interval of 40.94 ± 4.51 days. These images were all scanned on a SIEMENS TRIO 3T scanner in Beijing Normal University, all subjects signed an informed consent, and the data were well-anonymized before uploading. Structural MRI data were acquired using an MPRAGE sequence: 128 sagittal slices, repetition time (TR) = 2,530 ms, echo time (TE) = 3.39 ms, inversion time (TI) = 1,100 ms, slice thickness = 1.33 mm, field of view (FOV) = 256 × 256 mm^2^, and voxel size = 1.33 × 1.00 × 1.00 mm^3^. rs-fMRI data were obtained using an echo-planar imaging (EPI) sequence: 33 axial slices, TR = 2 s, TE = 30 ms, slice thickness = 3.5 mm, gap = 0.7 mm, FOV = 200 × 200 mm^2^, and voxel size = 3.1 × 3.1 × 4.2 mm^3^, with 200 dynamics in total. The current study only used fMRI and T1 data, and seven subjects whose T1 or BOLD data are incomplete were excluded. Therefore, 50 subjects were included finally (23.05 ± 2.29 years, male/female: 30/20). In the current study, the first scan was used for all of the analyses, and the second scan was only used in the test-retest reliability analysis.

#### Simulated Images

A simulation was performed based on the empirical rs-fMRI. The simulated images were generated from empirical rs-fMRI in individual space by the following processes: (1) For each voxel, a random white noise time course with same length (190 time points after removing the first 10) of empirical images was generated; (2) the signal intensity was normalized to maintain the mean intensity of the time course as constant value equal to 1,000, and tSNR equal to 100. These values were comparable to those in the brain regions of our empirical images. Therefore, the simulated images had constant tSNR value across different voxels, different subjects in the individual space, and in a same individual space to the corresponding empirical images.

### Data Processing

#### Preprocessing

The empirical fMRI images were preprocessed in DPABI (Yan et al., 2016). The pipeline procedure included the following steps: (1) discarding the first 10 volumes; (2) slice timing; (3) head motion correction; (4) regressing out the nuisance variables [including six head motion parameters and their derivatives, the average cerebrospinal fluid (CSF), white matter (WM) signal, and the linear term]. A band-pass filter (0.01–0.08 Hz) was additionally added for FC and ReHo but not for tSNR and ALFF.

#### Spatial Normalization Parameters

The structural T1-weighted images were coregistered to fMRI for each subject. Based on the coregistered structural images, normalization parameters were estimated using the unified segmentation (Ashburner and Friston, 2005) in SPM12 for each subject and each scan. These normalization parameters were used to normalize each fMRI image in each time point in Prenorm strategy as well as normalize the ALFF, ReHo, and FC maps calculated in individual space in Postnorm strategy. When normalizing these fMRI images, the voxel size in MNI space was set as 3 × 3 × 3 mm^3^, and the bounding box was set as [−90, −126, −72; 90, 90, 108]. We use this default setting of DPABI whose voxel size is comparable to those in individual space to reduce the differences of the voxel number before and after normalization. The interpolation was set as “Trilinear,” which is the default option in DPABI and in SPM.

#### tSNR, ALFF, and FC Calculations

We first calculated the tSNR, ALFF, ReHo, and FC in individual space. In each individual space, tSNR, ALFF, ReHo, and seed-based FC of default mode network (DMN) were calculated after the preprocessing. Specifically, the rs-fMRI time series in an individual space after preprocessing were translated to frequency domain by a fast Fourier transform, and the ALFF value was defined as the average square root across the low-frequency band of 0.01–0.08 Hz (Zang et al., 2007). The ReHo value in one voxel is defined as the Kendall coefficient of concordance among it and its 26 neighbor voxels (Zang et al., 2004; Zuo et al., 2013). For FC, the seed was defined as a 6-mm radius spherical region of interest (ROI) centered at [−5, −49, 40] in MNI space, located at precuneus (PCu), which is a classic seed ROI of DMN (Fox et al., 2005). To calculate in individual space, the seed ROIs were inversed normalized into each of the individual spaces, and then the FC value of each voxel was defined as the correlation between BOLD signal in this voxel and the average BOLD signal within the seed ROI.

tSNR, ALFF, ReHo, and FC in MNI space under two different strategies were then calculated. Under the Prenorm condition, the spatial normalization was applied to both simulated images and preprocessed empirical images. The tSNR, ALFF, ReHo, and FC maps were calculated for each subject in MNI space. For Postnorm, the tSNR, ALFF, ReHo, and FC in MNI space were generated by performing spatial normalization directly on tSNR, ALFF, ReHo, and FC maps in individual space.

For FC, a one-sample *t*-test was applied for both Prenorm and Postnorm. The regions showed significant positive results in either strategy (GRF correction by DPABI, voxel *p* < 0.01, cluster *p* < 0.05, compared to zero) and were defined as a DMN mask that is used to exclude the voxels with very low FC values, which is often not interested in rs-fMRI studies.

For simulated images, it was generated by replacing the time courses of the empirical fMRI. The spatial normalization of each corresponding subject can also directly be applied on the simulated images. Specifically, for Postnorm, the tSNR map was calculated in individual space and then transformed into MNI space. As expected, the tSNR value is constant among all voxels and subjects both before and after normalization. For Prenorm, the simulated images were first normalized and tSNR maps were calculated based on these normalization images. We did not calculate ALFF, FC, and ReHo for simulated images.

### Statistical Analysis

#### Simulated Images

The tSNR has exactly constant value and no intersubject variability in individual space. Therefore, we have “golden standard” to compare Prenorm and Postnorm for simulated images.

First, the mean tSNR value of the group for each voxel was calculated in MNI space for both Prenorm and Postnorm. A paired *t*-test was utilized to test the difference between these two strategies (GRF corrected, voxel level *p* < 0.01, cluster *p* < 0.05). However, it is worth to note that it is reasonable to expect that after Postnorm, tSNR maps would be constant maps as it is in individual space. In contrast, if Prenorm ones showed different results, it would be putatively caused by algorithmic effects.

Besides, we also focused on the intersubject variability. For each voxel within the brain mask in MNI space, coefficient of variation (CV, defined as STD divided by mean) across all subjects was used to quantify the intersubject variability of the tSNR. It can be expected that CV of Postnorm would be exactly zero, and if non-zero results were found in Prenorm condition, it may indicate that Prenorm can induce the algorithmic intersubject variability.

#### Empirical Images

tSNR, ALFF, ReHo, and FC maps for empirical rs-fMRI data were also compared between Prenorm and Postnorm. Similar to the analysis for simulated images, the mean tSNR/ALFF/FC/ReHo maps was calculated in MNI space for both Prenorm and Postnorm. A paired *t*-test was utilized to test how much difference is there between these two strategies (GRF corrected, voxel level *p* < 0.01, cluster *p* < 0.05). CV maps of tSNR, ALFF, ReHo, and FC were also generated and compared by a difference map between Prenorm and Postnorm.

Unlike simulated images, it is hard to define a golden standard for empirical images (e.g., larger CV is better or worse). However, given that the simulated images were generated from empirical images, we used a simulation–empirical correlation to demonstrate if the possible bias that affected simulated images can also explain the variation in empirical fMRI. Specifically, a Pearson correlations analysis was applied between the tSNR values of simulated images and rs-fMRI indices including tSNR, ALFF, ReHo, and FC of empirical images in MNI space for each voxel within the brain mask (GRF corrected, voxel level *p* < 0.01, cluster *p* < 0.05).

#### Test–Retest Reliability

As mentioned, the two separate scans of empirical fMRI images with the time interval 40.94 ± 4.51 days were acquired, which enabled us to investigate how normalization strategies impact the test–retest reliability. The between scans test–retest reliability of tSNR, ALFF, ReHo, and FC was investigated, and the intraclass correlation coefficients [ICC, defined as Eq. (1), where *Vb* and *Vw* are between-subject and within-subject variability, respectively] were calculated in each voxel for tSNR, ALFF, ReHo, and FC maps between two serial scans. Histogram was generated for the whole brain as well as gray matter regions (generated by segmentation and group mean gray matter probability larger than 0.4), and for FC, also in DMN mask. A paired *t*-test was utilized to validate if the ICC is different between the two normalization strategies for each of the rs-fMRI indices.

(1) $$\text{ICC}=\frac{V\,b-V\,w}{V\,b+V\,w}.$$

All of the statistical analyses in the current study were carried out by Matlab and AFNI (Cox, 1996). Results were represented by AFNI (Cox, 1996).

## Results

### Simulated Images

**FIGURE 1:**
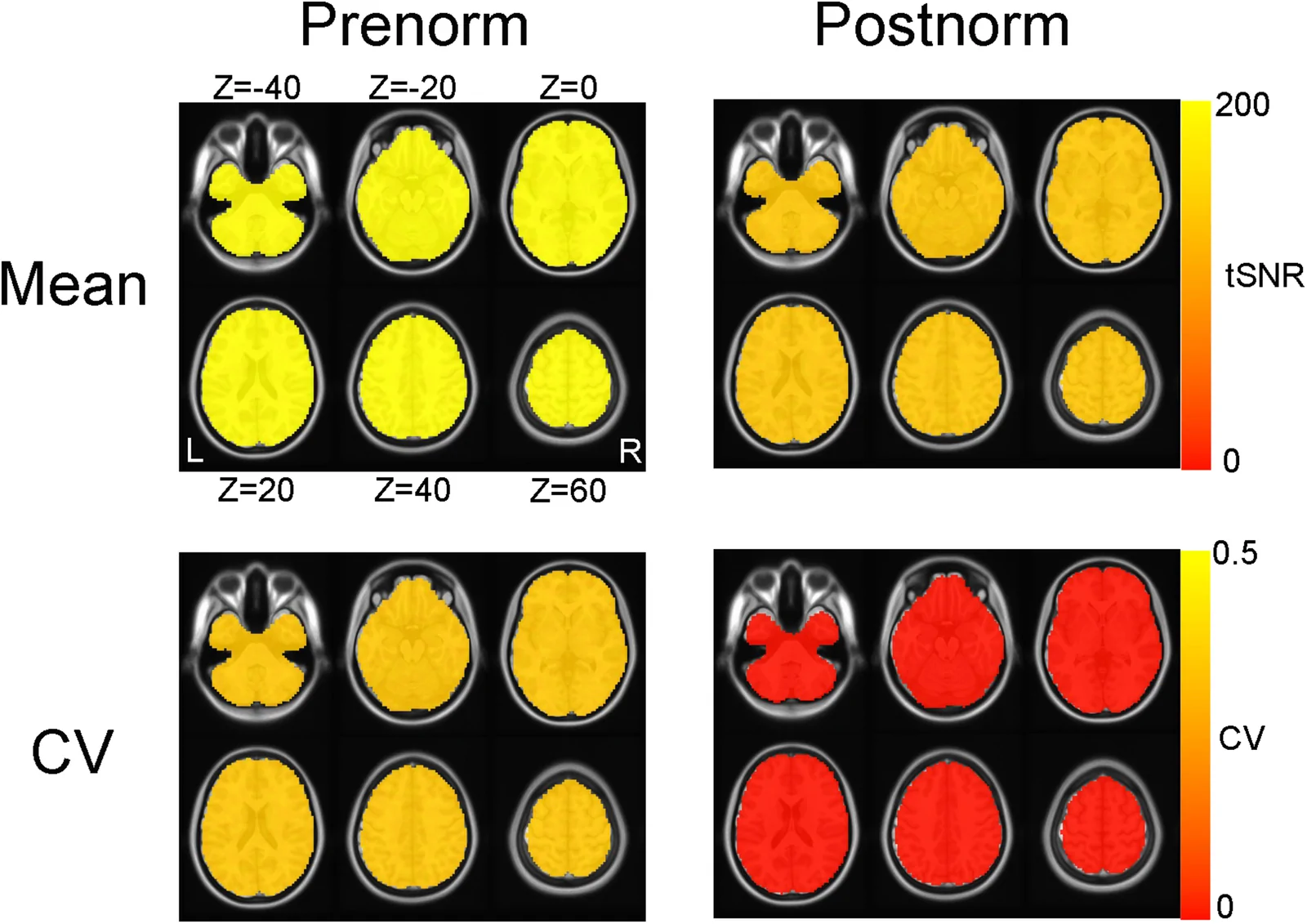
Effect of normalization strategies on temporal signal-to-noise ratio (tSNR) of simulated images. The group mean maps of tSNR and coefficient of variation (CV) maps of tSNR were shown for both normalization strategies. Both mean and CV maps for Postnorm are constant maps. For Prenorm, both mean and CV of tSNR were larger than Postnorm condition and are not constant but very limited spatial variation (so seem like constant maps too).

The effects of normalization strategies on tSNR in simulated images were shown in Figure 1. As expected, when using Postnorm strategy, the tSNR in MNI space kept exactly 100 as it is generated in individual space. In contrast, the mean tSNR values in each voxel is increased from 100 to near 200 after normalization for all voxels when Prenorm is utilized. The whole brain mean is 194.26 ± 1.17 after normalization, significantly larger than 100 according to a one-sample *t*-test, *t* = 635.90, *p* < 10^–97^.

For intersubject variability, after Prenorm, CV was consistently at a level above zero for all of the voxels (0.195 ± 0.075, lowest 1% quantile 0.135). In contrast, for Postnorm condition, CV across subject is exactly zero.

### Empirical Images

**FIGURE 2:**
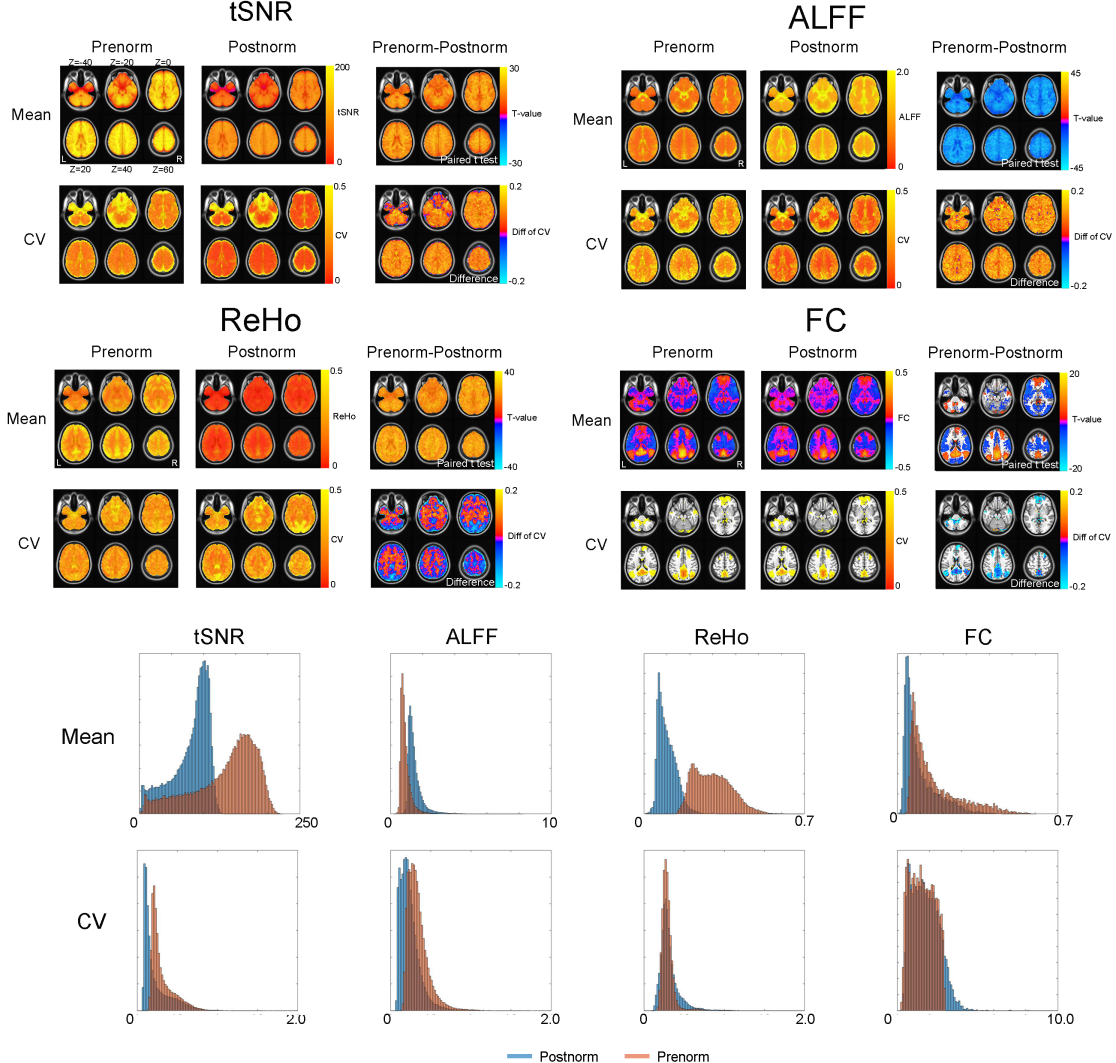
Effect of normalization strategies on temporal signal-to-noise ratio (tSNR), amplitude of low-frequency fluctuation (ALFF), regional homogeneity (ReHo), and seed-based functional connectivity (FC) of empirical images. The group mean maps of four rs-fMRI indices including tSNR, ALFF, ReHo, and seed-based FC [seed coordinate (–5, –49, 40)] were shown for both normalization strategies. A paired *t*-test (GRF corrected, voxel *p* < 0.01, cluster *p* < 0.05) was utilized to compare them between Prenorm and Postnorm. The coefficient of variation (CV) of each index under each normalization strategy was also shown and compared by the difference map. For FC, given that a lot of regions have negative or very small FC values, CV is not well defined, therefore we only show the CV within a default mode network (DMN) mask and the histogram for FC is within the DMN mask. The bottom part showed the histogram of tSNR, ALFF, ReHo, and FC as well as their CV for both normalization strategies. Again, the histograms of tSNR, ALFF, and ReHo were for whole brain, but the histogram of FC is for the DMN mask.

The difference between Prenorm and Postnorm strategies on tSNR, ALFF, ReHo, and FC in empirical images was shown in Figure 2. Compared to Postnorm, Prenorm have significantly lower ALFF but higher tSNR and ReHo values in all of the brain voxels. For FC, Prenorm have higher FC value in most of DMN regions and lower FC value in the regions of task-positive networks compared to Postnorm (paired *t*-test, GRF correction, voxel *p* < 0.01, cluster *p* < 0.05).

For intersubject variability, after Prenorm, CVs of tSNR and ALFF were both larger compared to those of Postnorm. For ReHo, the regions in WM showed that CV after Prenorm was larger than that after Postnorm but an inverse result in gray matter and edge of the brains. For FC, given that lots of brain regions have very FC values near zero, the CV would tend to be infinite and badly defined. Therefore, we only focused on the voxel within the DMN mask. In this region, CV was low after Prenorm compared to that after Postnorm.

### Correlation Between Simulated and Empirical Images

The increase of tSNR as well as its CV in empirical data after Prenorm compared to those after Postnorm was consistent with the simulation. Further correlation analysis investigated how and if the effect that Prenorm can induce intersubject variation found in simulation can explain the results from empirical images.

**FIGURE 3:**
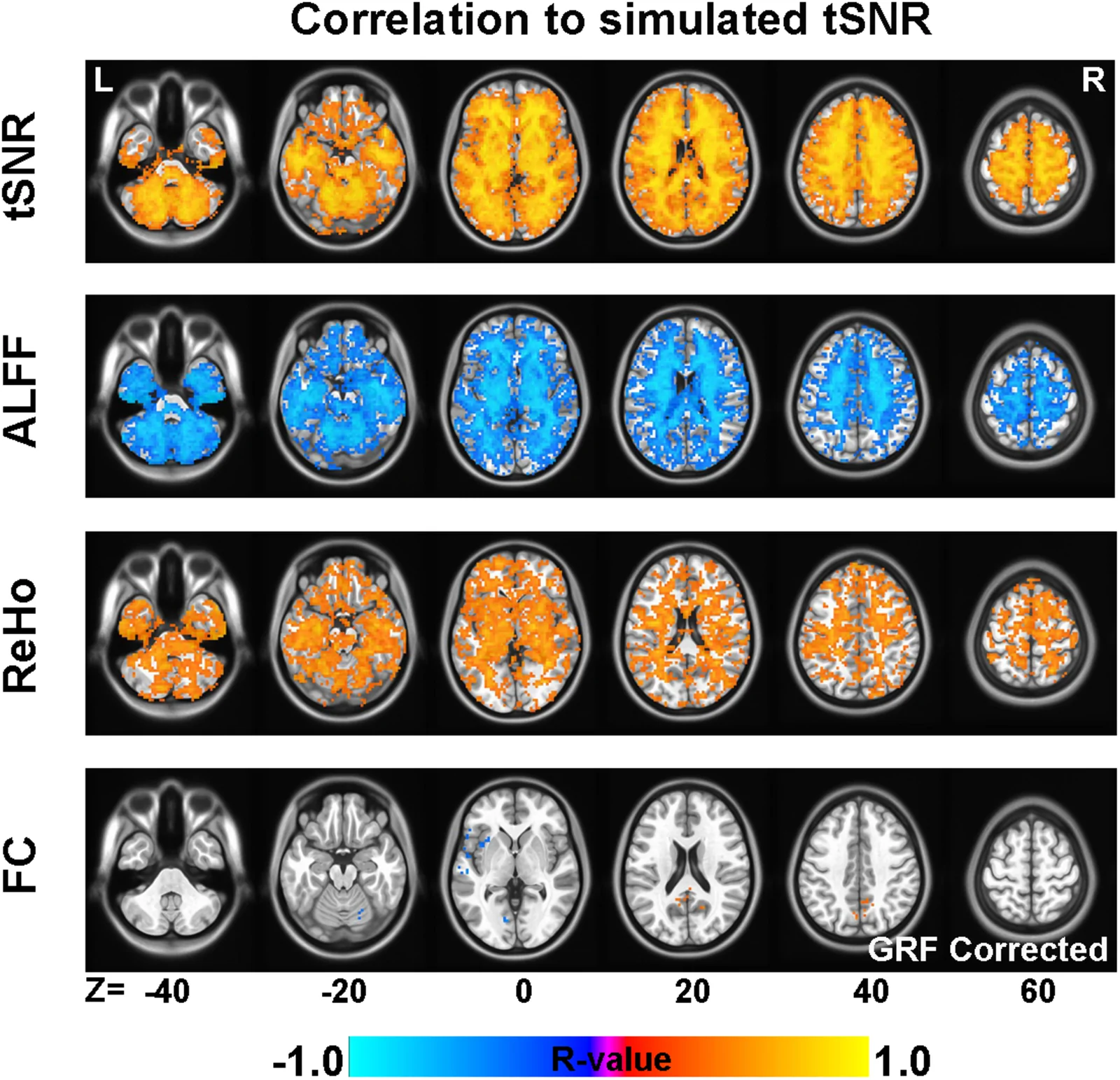
The correlation between temporal signal-to-noise ratio (tSNR) of simulated images and fMRI indices of empirical images. The across-subject correlation between tSNR of simulated images and empirical fMRI indices, including tSNR, amplitude of low-frequency fluctuation (ALFF), regional homogeneity (ReHo), and functional connectivity (FC) (GRF corrected, voxel *p* < 0.01, cluster *p* < 0.05).

As illustrated in Figure 3, tSNR in simulated images was significantly correlated with tSNR, ALFF, and ReHo of empirical images when Prenorm strategy is used. The empirical tSNR showed positive correlation (GRF corrected, voxel level *p* < 0.01, cluster *p* < 0.05) with simulated tSNR in all of the brain regions, especially in WM. The regions that showed lower correlation were on the edge regions and the base of the brain. Similar patterns were found for the correlation maps between empirical ALFF and simulated tSNR, except that the correlation is negative. For ReHo, there are large regions showing significant correlation with simulated tSNR in WM. For FC, only a small region near the seed ROI showed a significant positive correlation, and voxels around the left insula regions showed a significant negative correlation between FC and simulated tSNR (GRF corrected, voxel level *p* < 0.01, cluster *p* < 0.05).

### Test–Retest Reliability

**FIGURE 4:**
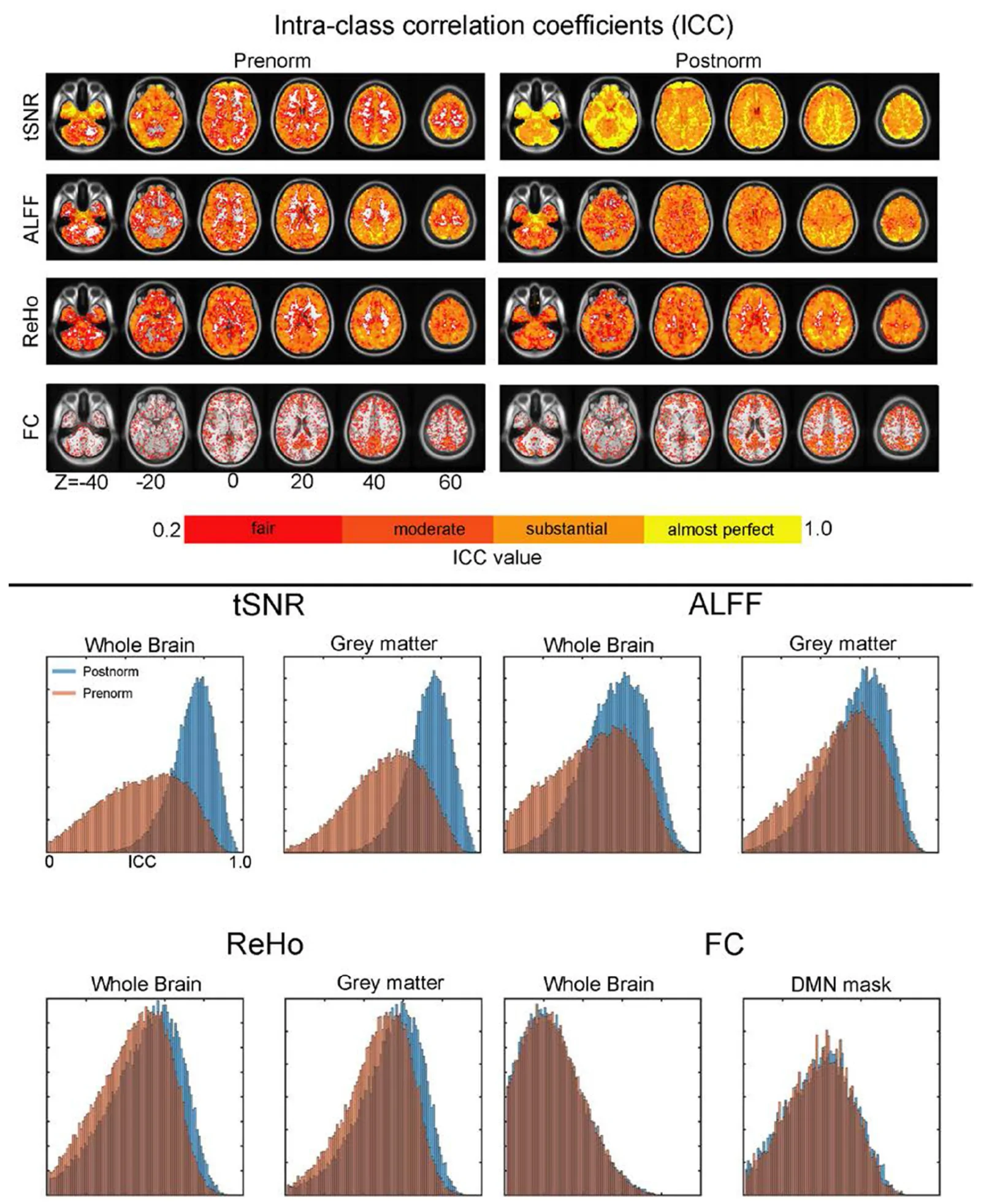
Effect of normalization strategies on intraclass correlation coefficients (ICCs) of the amplitude of low-frequency fluctuation (ALFF), regional homogeneity (ReHo), and functional connectivity (FC). **(Upper)** The left column showed the ICC values in each voxel after Prenorm and the right column for Postnorm. Each row for temporal signal-to-noise ratio (tSNR), ALFF, ReHo, and FC, respectively. The “fair reliability” mean means 0.2 < ICC < 0.4, “moderate” means 0.4 < ICC < 0.6, “substantial” means 0.6 < ICC < 0.8, and “almost perfect” means ICC > 0.8. **(Bottom)** Histogram of ICC. For each rs-fMRI index, the histograms of ICC for both normalization strategies were shown. For tSNR, ALFF, and ReHo, histograms within whole brain and gray matter regions were illustrated. For FC, besides whole brain, we focused on default mode network (DMN) regions to exclude regions with the very small FC values, as well as the controversial negative FC regions. According to a paired *t*-test, ICC of tSNR, ALFF, and ReHo is significantly higher when Postnorm is used compared to those when Prenorm is used. In contrast, FC showed no significant difference.

The test–retest reliability of the rs-fMRI features between the two scans was measured using ICC, and the results were illustrated in Figure 4. Generally, tSNR, ReHo, and ALFF showed quite similar results: ICC is higher when Postnorm is utilized compared to Prenorm. Some of the WM and subcortical regions have quite poor reliability (ICC < 0.2) when Prenorm was applied compared to those at least fair (ICC > 0.2) when applying Postnorm. The histogram showed that the ICC distribution within the whole brain and within gray matter regions of tSNR for Postnorm is skewed to the higher end compared to those of Prenorm. Similar results were found for ALFF and ReHo. Paired *t*-test showed that Prenorm ICC values were significantly higher than Postnorm ICC for tSNR, ALFF, and ReHo (*p* < 0.00001, note sample size is voxel number and therefore very huge). In contrast, the FC showed very similar ICC values and distributions between the two normalization strategies.

## Discussion

In the current study, we have demonstrated: (1) Prenorm strategy can change the tSNR values and induce intersubject variability from simulated images with constant tSNR. (2) The features of empirical fMRI including tSNR, ALFF, ReHo, and FC would be different between Prenorm and Postnorm. Furthermore, the intersubject variability of these were also different between normalization strategies. (3) Importantly, the algorithmically induced intersubject variation of tSNR under Prenorm in simulated images can explain the intersubject variation in empirical results of tSNR, ALFF, and ReHo. (4) The ICC values of tSNR, ALFF, and ReHo for Prenorm are significantly lower than those of Postnorm.

It is not a surprise that spatial normalization can increase the tSNR compared to it in individual space. A previous study indicated that resampling and interpolation in both Prenorm and Postnorm may superimpose spatial autocorrelation on fMRI data (Wu et al., 2011). This “additional smoothing” may suppress the noise contribution in fMRI signal (Krüger and Glover, 2001) and directly lead to increased tSNR. In our simulation, we use white noise with constant tSNR and found that Prenorm increased tSNR value while Postnorm did not. This result indicated that Postnorm, which does interpolation on scalar values, may have less effect than Prenorm, which interpolated the time courses. The results in empirical images were very consistent with this assumption. Prenorm has higher tSNR, therefore less total variation, which leads to smaller ALFF, but higher correlation both among local voxels (ReHo) and between distinct regions (FC), as illustrated in Figure 2.

However, most rs-fMRI studies are interested in the intersubject difference rather than the value of rs-fMRI indices. Given that there is no individual difference and no neural signals in our simulated images, the intersubject variability after Prenorm in simulated images was putatively induced by algorithmic reasons. We suspected that it may be induced by the fact that the extent of the deformation during spatial normalization varied across subjects, which can determine the extent of resampling and interpolation. Interpolation can increase tSNR, and therefore intersubject difference of interpolation directly leads to intersubject variability of tSNR. Again, this effect in Postnorm seems weaker, which has no effect in simulation (resampling would not change constant), and smaller effect in empirical images (CV of tSNR is less in Postnorm than that in Prenorm). Consistent result was found for CV of ALFF, which is larger in Prenorm. However, ReHo and FC showed different results. This is possibly caused by increased FC and ReHo values in Prenorm compared to those in Postnorm, especially in DMN for FC and in all gray matter regions for ReHo. This effect may overwhelm the effect of increased intersubject variability of the noise level. Therefore, when in WM where there may be less neural signals, ReHo showed increased CV in Prenorm than that in Postnorm, which is consistent with tSNR. It is also worth to note that ReHo is differently defined in Prenorm and Postnorm given the range of “neighbor voxels” that are actually different; this may lead to these complex differences (Zuo et al., 2013).

Furthermore, Prenorm tSNR in empirical images is highly correlated across subjects with Prenorm tSNR in simulated images, indicating that the algorithmic effect in simulated images can also explain the intersubject variability of tSNR in empirical fMRI. Moreover, similar widespread significant correlation was found between simulated tSNR and empirical ALFF and ReHo. The negative correlation for ALFF is expected since higher tSNR means lower total variation, which can be reflected by ALFF (Garrett et al., 2013). These results further indicated that there is a significant portion (for the significant regions in Figure 3, *R*^2^ > 0.1) of the intersubject variability of ALFF and ReHo that can be explained by pure algorithmic effect of resampling and interpolation during normalization if Prenorm is used. Given that most rs-fMRI studies focused on between-subject differences, this indicated that for ALFF and ReHo, the Prenorm strategy may need to be avoided.

Consistent with the algorithmic induced intersubject variability, ICC between two serial scans further suggested that using Prenorm would decrease the test–retest reliability of rs-fMRI features compared to Postnorm. Less reliability may lead to less statistical power, low reproducibility, and demand of big data size (Button et al., 2013; Poldrack et al., 2017; Hedge et al., 2018) and has been recently highlighted as a challenge in fMRI field (Bennett and Miller, 2010; Xing and Zuo, 2018; Zuo et al., 2019a, b). Therefore, our findings strongly suggested that all of the rs-fMRI studies using ALFF and ReHo in the future use Postnorm instead of Prenorm. Although ICC of FC seems to be not sensitive to the normalization strategy compared to ALFF and ReHo, given that the Prenorm can induce variability of tSNR and the fact that noise level can affect FC values (Duff et al., 2018), FC studies also should choose normalization strategies with caution. Also, given that the increased variability of fMRI features may be caused by the geometrical difference during normalization, special caution should be taken with spatial normalization when investigating the functional–structural relationship (Di et al., 2013; Qing and Gong, 2016) or investigating the population that may have various degrees of brain atrophy, like dementia (Qing et al., 2017; De Vos et al., 2018). It is also worth noting that the effect of Prenorm is more severe in subcortical and WM regions. Therefore, studies that focus on WM BOLD signal and subcortical spontaneous activity should be more cautious with choice of spatial normalization strategy or just avoid spatial normalization (Makedonov et al., 2013, 2016; Ji et al., 2017; Qing et al., 2017).

It is worth to note that the effect of normalization may be related to the interpolation methods. In the current study, we use the “trilinear” interpolation because it is the default setting of most fMRI data processing toolboxes, such as FSL (“fnirt”), SPM (Normalization-Write), and AFNI (“3dAllineate”), and therefore has been widely applied. We also repeated our analysis using “nearest neighbor,” fourth and seventh B-spline interpolation options, which were provided in SPM. When using “nearest neighbor,” exactly the same ALFF and tSNR would be found for Prenorm and Postnorm, but there were slight differences for ReHo and FC, which may be caused by different definitions of neighbor voxels and seed time course in different strategies (details can be found in the Supplementary Material). This validated that difference effects of interpolation on time course and scalar values lead to our finding of the difference between Prenorm and Postnorm. For higher order interpolation, such as fourth or seventh B-spline, most of our findings still showed similar and significant results compared to “trilinear” but with more conservative effect size (however, some results were inversed, see the Supplementary Material). However, using nearest neighbor may decrease smoothness of the results, and it is time-consuming to use higher order interpolation, especially when treating large data cohorts. Additionally, other type of interpolation like using sinc function instead of linear may also have an impact but was more rarely be used during spatial normalization (Strother et al., 2004). At last, when large kernel spatial smoothing was done, the additional interpolation among voxels would attenuate the effect of interpolation during normalization, and therefore we did not include and discuss this step for simplicity.

There are some limitations of the current study. First, we only use the unified segmentation (Ashburner and Friston, 2005) to perform the normalization. Although we believe that the effect of Prenorm on temporal features is independent to the different estimating methods of spatial transformation, the finding in the current study still need to be validated against other normalization algorithms, such as DARTEL in SPM (Ashburner, 2007), utilization of the EPI templates instead of structural templates (Calhoun et al., 2017), and also other toolboxes, such as FSL (Jenkinson et al., 2012). Moreover, our results were based on a relatively small publicly available dataset that only included 50 subjects, which all have the same scanning parameters and using the same scanner. The current findings need further validation across various types of scanners, various image resolutions, and a larger dataset in the future.

## Conclusion

In conclusion, the current study demonstrated that the commonly used spatial normalization, Prenorm, may induce an artificial variability across subjects on tSNR of rs-fMRI and significantly contribute to the intersubject variability of ALFF and ReHo and reduce their reliability compared to Postnorm. These results suggest that future fMRI studies using ALFF and ReHo should perform spatial normalization with caution and suggest Postnorm as an improvement.

## Data Availability Statement

Publicly available datasets were analyzed in this study. This data can be found here: http://fcon_1000.projects.nitrc.org/indi/CoRR/html/.

## Ethics Statement

All images of the human subjects used in the current study are downloaded from the public dataset “Consortium for Reliablity and Reproducibility,” published by Zuo et al. (2014) and all subjects are anonymous. The download website can be found at http://fcon_1000.projects.nitrc.org/indi/CoRR/html/_static/downloads.html.

## Author Contributions

ZQ, BZG, and BZU designed the study. ZQ wrote the manuscript. ZQ, XZ, MY, SW, and XW performed the data management, data analysis, and artworks. ZQ, BZG, ZN, and JH decided the manuscript structure and logic. ZN and JH improved the language expression.

## Conflict of Interest

The authors declare that the research was conducted in the absence of any commercial or financial relationships that could be construed as a potential conflict of interest.
