# Supplementary material for: The Impact of Spatial Normalization Strategies on the Temporal Features of the Resting-State Functional MRI: Spatial Normalization Before rs-fMRI Features Calculation May Reduce the Reliability
Source: Front Neurosci. 2019 Nov 26;13:1249. doi: 10.3389/fnins.2019.01249 (PMC6902012; doi:10.3389/fnins.2019.01249)
Supplement: Supplementary file 1 [file Table_1.DOCX]

Supplementary table. The effect of interpolation methods

|  |  | Mean | | | | CV | | | | ICC | | | |
| --- | --- | --- | --- | --- | --- | --- | --- | --- | --- | --- | --- | --- | --- |
|  |  | NN | TL | 4^th^ | 7^th^ | NN | TL | 4^th^ | 7^th^ | NN | TL | 4^th^ | 7^th^ |
| tSNR | Prenorm | 79.26±29.92 | 132.94±50.0 | 88.33±33.46 | 84.14±31.95 | 0.31±0.25 | 0.31±0.16 | 0.29±0.21 | 0.29±0.22 | 0.49±0.15 | 0.48±0.22 | 0.62±0.15 | 0.65±0.14 |
|  | Postnorm | 79.26±29.92 | 79.09±29.46 | 79.48±30.28 | 79.49±30.31 | 0.31±0.25 | 0.23±0.18 | 0.29±0.22 | 0.29±0.23 | 0.49±0.15 | 0.71±0.18 | 0.68±0.13 | 0.67±0.14 |
| ALFF | Prenorm | 1.47±0.52 | 0.92±0.35 | 1.33±0.50 | 1.39±0.51 | 0.31±0.18 | 0.34±0.15 | 0.30±0.16 | 0.29±0.16 | 0.37±0.18 | 0.42±0.24 | 0.51±0.20 | 0.51±0.20 |
|  | Postnorm | 1.50±0.54 | 1.49±0.51 | 1.50±0.56 | 1.50±0.56 | 0.31±0.18 | 0.24±0.14 | 0.30±0.16 | 0.31±0.16 | 0.37±0.18 | 0.54±0.19 | 0.52±0.19 | 0.51±0.20 |
| ReHo | Prenorm | 0.15±0.05 | 0.30±0.08 | 0.18±0.06 | 0.17±0.06 | 0.38±0.13 | 0.29±0.07 | 0.33±0.12 | 0.33±0.13 | 0.35±0.20 | 0.44±0.19 | 0.53±0.19 | 0.54±0.19 |
|  | Postnorm | 0.10±0.04 | 0.10±0.04 | 0.10±0.04 | 0.10±0.04 | 0.39±0.23 | 0.32±0.14 | 0.36±0.18 | 0.37±0.18 | 0.38±0.20 | 0.47±0.21 | 0.45±0.21 | 0.45±0.21 |
| FC | Prenorm | 0.08±0.09 | 0.16±0.11 | 0.09±0.09 | 0.08±0.09 | 1.70±0.61 | 1.57±0.72 | 1.65±0.63 | 1.67±0.62 | 0.26±0.17 | 0.37±0.18 | 0.38±0.19 | 0.38±0.19 |
|  | Postnorm | 0.10±0.08 | 0.10±0.07 | 0.10±0.08 | 0.10±0.08 | 1.77±0.66 | 1.61±0.71 | 1.74±0.69 | 1.75±0.68 | 0.23±0.17 | 0.37±0.18 | 0.35±0.20 | 0.34±0.20 |

This table showed the results of the difference between Prenorm and Postnorm when different interpolation methods were used during spatial normalization. NN means “nearest neighbor”, TL means “trilinear” which is used in the main body of our paper, 4^th^ and 7^th^ means 4^th^/7^th^ B-spline.

For NN, ALFF and tSNR is exactly same between Prenorm and Postnorm, but FC and ReHo showed some difference. This can be expected because that the spatial transformation is same. For one MNI voxel, the nearest voxel in individual space is the same no matter Prenorm or Postnorm is performed. Therefore, there would be no difference between "directly write that individual voxel's time course on this MNI voxel, and then calculated ALFF/tSNR" and “directly write the ALFF/tSNR of that individual voxel on this MNI voxel". However, for ReHo and FC, it is more complex because FC and ReHo in one voxel is not only depended on the time course in this voxel. For ReHo, the neighbor voxels would be not identically defined in individual and MNI space. For FC, the seed time course would be differently calculated in individual and MNI spaces. This leads to different results.

The difference of mean, CV and ICC between Prenorm and Postnorm were all significant given the huge sample size for higher order B-spline. It is worth to note that most of the results have some direction but smaller effect size than those using trilinear option. Besides, there is some inversed results, for example, ICC is higher under Prenorm than Postnorm for ReHo and FC when higher order B-spline interpolation is used, especially for ReHo. However, a higher order interpolation may increase spatial autocorrelation of fMRI data which may contaminate the real local homogeneity of brain activity. Therefore we still would not suggest to use such interpolation in ReHo study.
